# Genetics of Keratoconus: A Comprehensive Review

**DOI:** 10.3390/genes16101147

**Published:** 2025-09-27

**Authors:** Raul Hernan Barcelo-Canton, Darren S. J. Ting, Jodhbir S. Mehta

**Affiliations:** 1 Tecnologico de Monterrey, School of Medicine and Health Sciences, Institute of Ophthalmology and Visual Sciences, Monterrey 66278, Mexico; 2 Academic Unit of Ophthalmology, Department of Inflammation and Ageing, College of Medicine and Health, University of Birmingham, Birmingham B15 2TT, UK; 3 Birmingham and Midland Eye Centre, Sandwell and West Birmingham NHS Trust, Birmingham B18 71H, UK; 4 Academic Ophthalmology, School of Medicine, University of Nottingham, Nottingham NG7 2RD, UK; 5 Ophthalmology & Visual Sciences Academic Clinical Programme (EYE-ACP), Duke-NUS Medical School, Singapore 169857, Singapore; 6 Singapore Eye Research Institute, Singapore 169856, Singapore; 7 Singapore National Eye Centre, Singapore 168751, Singapore

**Keywords:** candidate gene, genes, genetic association, keratoconus, loci

## Abstract

Keratoconus (KC) is a progressive, multifactorial corneal ectatic disorder characterized by localized stromal thinning and irregular astigmatism, with incidence and prevalence varying markedly among populations. These differences are influenced by environmental exposures, behavioral factors, and genetic predisposition. A positive family history is a well-established high-risk factor, and KC has also been documented in association with syndromic disorders such as Down syndrome, connective tissue disorders, and certain metabolic diseases. Over the past decades, numerous candidate genes have been investigated, encompassing those involved in extracellular matrix (ECM) assembly, collagen synthesis and cross-linking, oxidative stress defense, wound healing, and transcriptional regulation. Modern genomic approaches, including genome-wide association studies (GWAS), linkage analyses, and next-generation sequencing, have identified multiple loci and variants with potential pathogenic roles. Nonetheless, several genes have also been systematically tested and found to show no association in specific populations, highlighting the genetic variability of KC and the potential influence of population-specific factors. This dual landscape of positive and negative genetic findings underscores the complexity of KC pathogenesis and the necessity for ethnically diverse cohorts. In this review, we synthesize current evidence on genes implicated in KC, integrating confirmed pathogenic variants, associations, and negative findings across diverse populations, to provide a comprehensive overview of the genetic architecture of KC and to outline priorities for future research aimed at improving diagnosis, risk stratification, and therapeutic development.

## 1. Introduction

The cornea is a transparent, avascular tissue forming the anterior-most part of the eye, accounting for two-thirds of its total refractive power [1,2]. Structurally, it is composed of five organized layers that maintain its transparency and biomechanical strength, essential for clear vision. Its ECM, primarily composed of collagen and proteoglycans, ensures both optical clarity and structural integrity under constant intraocular pressure [1,3]. The cornea also serves as a barrier against environmental insults while participating actively in wound healing and maintaining its curvature [2]. Disruptions in corneal structure and biomechanics can lead to ectatic disorders such as KC.

KC has been described as the most common primary ectatic disease of the cornea [4,5]. It is a bilateral, asymmetric disease which can lead to a progressive thinning and steepening of the cornea, can induce irregular astigmatism, and often induces a decrease in visual acuity [6]. If the disease progresses, the cornea’s central thinning and protrusion can lead to severe complications which can affect eyesight, including scarring and edema. In 2015, the first global consensus on KC, established by an international panel of experts, defined the condition by the presence of three mandatory diagnostic features: abnormal posterior corneal ectasia, irregular corneal thickness distribution, and clinically non-inflammatory corneal thinning [7]. It typically affects young adults around the second decade and usually progresses until the 3rd decade [5].

**Figure 1:**
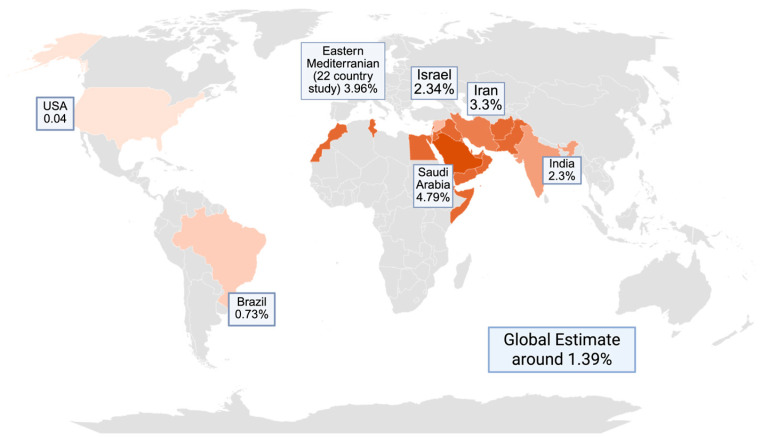
Prevalence of KC amongst various countries.

The prevalence of KC shows wide variation between populations, with significant differences across ethnic groups and geographic regions, although consistent gender-based disparities have not been observed [7,8]. This variability can be attributed to the methods for prevalence investigation but also to probable genetic and environmental differences between populations. Among Middle Eastern countries, the prevalence of KC is reported to be high. Iran reports a prevalence of around 3.3%, while Saudi Arabia has reported an estimated prevalence of 4.79% among pediatric groups [9,10]. Israel reported an estimated prevalence of 2.34% [11]. In Denmark, the estimated prevalence of KC has been reported as 44 cases per 100,000 individuals in the general population [12]. In India, the prevalence of KC has been estimated at approximately 2.3% among individuals aged 30 years residing in central regions of the country [13]. Syria reported a prevalence of 1.43% among university students [14]. Another study of 22 countries in the eastern Mediterranean estimated the prevalence of KC at 3.96% [15]. Brazil reports a prevalence of 0.73% among high school students [16]. In the United States, the prevalence was estimated in 2019 to be around 0.04% [17]. A global meta-analysis estimated the global prevalence of KC at 1.39 per 1000 patients [18]. Figure 1 visually examines the prevalence of KC amongst various countries. Overall, the reported prevalence of KC demonstrates considerable variability across different regions worldwide.

KC is considered a multifactorial disease, involving a variety of genetic predispositions, environmental exposures and biomechanical stress [19]. Central mechanisms to the pathogenesis of this disease include the ECM degradation and weakening, abnormalities of corneal collagen cross-linking, oxidative stress, and direct microtrauma, mainly in the form of eye rubbing [19,20]. Environmental and host diseases such as atopy act as external accelerators of these processes, thus aggravating molecular vulnerabilities [21]. Genetic predisposition mixed with these central pathophysiological mechanisms leads to the formation of KC.

A range of risk factors have been identified as potential contributors to the development and progression of KC. Genetic, ethnic, environmental, and mechanical factors have all been shown to influence both the development and pathogenesis of KC [7,22]. Alterations in corneal stromal collagen, primarily driven by changes in ECM stiffness and dysregulation of biochemical signaling, lead to a loss of structural integrity and distortion of the corneal architecture [23].

Regarding the genetic implications in KC, a positive family history is recognized as a significant risk factor, and numerous genes have been implicated in both the pathogenesis and predisposition to the disease [24]. Advances in genetic research on KC have led to the identification of associated genes and loci, aided by GWAS, structural DNA variant analysis, and exome/genome sequencing [22,25]. This article aims to review the genetics and advancements in genetic studies for KC and to highlight current research and future directions for the pathogenesis of KC.

## 2. Understanding the Different Types of Genetic Studies

### 2.1. Candidate Genes

KC is considered to be a genetically heterogeneous disease, as multiple genes, loci, and alleles can contribute to the disease pathogenesis [26]. A candidate gene is one that is hypothesized to be involved, either directly or indirectly, in the development of a disease, typically based on prior biological knowledge, functional relevance, or the presence of disease-associated mutations. Such genes are often expressed in affected tissues, located in chromosomal regions linked to the disease, or involved in critical biological pathways underlying the condition [27]. Up to 35 candidate genes have been identified and associated with KC, mainly via linkage studies [28]. Candidate genes are usually the first step when investigating genes and relationships with diseases.

### 2.2. Linkage Studies

Genetic linkage studies aim to identify the approximate chromosomal regions that may harbor genes contributing to the development of specific diseases. These studies rely on the analysis of hereditary patterns within families, evaluating how frequently genetic markers are passed on with the disease phenotype. Linkage analysis is particularly effective in the context of Mendelian disorders, where inheritance patterns are more clearly defined [29,30].

### 2.3. Genome Wide Association Studies (GWAS)

GWAS aim to prove a genetic correlation between altered genomic loci and traits from specific diseases [31]. More specifically, they tend to identify single nucleotide polymorphisms (SNPs) by examining the entire genome in a group of the population large enough to determine a specific link by using a case/control system [32]. GWAS have been increasingly used in KC in the last decade and have helped deepen the initial relationship of diverse genes in a more advanced setting, aiming to provide future therapeutic advances [30].

### 2.4. Genetic Expression Tests

Gene expression tests specifically evaluate the transcriptional activity of genes by measuring RNA expression levels. These analyses can reveal the upregulation or downregulation of specific genes in disease compared to healthy conditions, thereby uncovering dysregulated molecular pathways and cellular functions involved in pathogenesis [33].

## 3. Genetic Affections in KC

### 3.1. Familial KC

**Figure 2:**
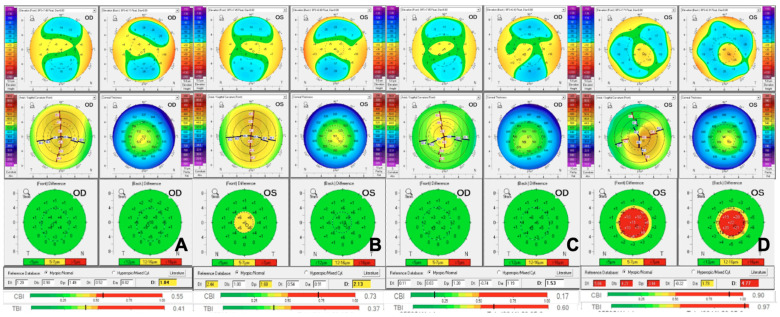
Brother and sister with a family history of KC. **Note:** Belin Ambrosio Display (BAD): Analysis for KC and ectasia, which studies the relationship between pachymetry and elevation maps. Corvis Biomechanical Index (CBI): Biomechanical analysis of the corneal response to an air puff stimulus. Tomography and Biomechanical Index (TBI): A combined index which integrates tomographic data with the biomechanical information from CBI and analyzes it against a population-specific database. (**A**) Right eye of first sibling (female, 18) and (**B**) left eye, both with normal tomography but abnormal biomechanics. (**C**): Right eye of the older second sibling (male, 23) with a normal corneal tomography and topography but abnormal corneal biomechanics. His left eye (**D**) shows abnormalities at the BAD and biomechanics.

KC has long been recognized as a familial disease, with strong evidence supporting a genetic predisposition within affected families [26]. This familial pattern is further reinforced by studies on first-degree relatives, which provide additional insight into the heritable nature of the disease and its potential impact on clinical presentation. The presence of a first-degree relative with KC is a well-established risk factor for developing the disease. Earlier estimates suggest a positive family history in around 13.5% of patients [34]. Among 1226 first-degree relatives from the United States, the prevalence of KC was 3.3%, estimated to be 15–67 times higher than the average population [35]. In a recent study by Lapeyre and colleagues, the correlation of KC among first-degree relatives was estimated at 0.55 between parents, 0.29 between parents and offspring, and 0.49 among siblings, with an average rate of 9.05% of patients showing KC findings. These findings underscore the significant familial component and genetic predisposition associated with KC [36]. Another study in China determined that, of 661 first-degree relatives, an average of 8.8% were diagnosed with KC, with up to 29% showing suspect topographic changes [37]. A prospective Israeli study of 56 patients demonstrated around 18% of first-degree relatives were diagnosed with KC or as suspects [38]. While earlier studies suggested that a family history of KC does not correlate with disease severity, more recent research indicates that patients with a positive family history may exhibit greater disease severity when classified by the Amsler–Krumeich grading system [39,40]. More information is required to reach a relationship between disease severity and family history in KC patients. Figure 2 describes a case of siblings patients with a positive KC family history. 

Twin studies have confirmed the relationship of both monozygotic and dizygotic twins with KC. Although monozygotic twins exhibit a stronger concordance in disease presentation compared to dizygotic twins, both groups represent significant risk factors for the development of KC [41]. Discordance is still possible, as reported in other cases, most likely explained by the influence of environmental and behavioral risk factors [42]. Overall the concordance rate among monozygotic twins is around 54%, further supporting the genetic factor in the development of KC [43].

It is imperative to understand that while familial KC poses a higher risk to the development of this disease, familial penetrance and incomplete inheritance play major roles in the pathogenesis of the disease. While KC is inherited mainly by autosomal dominant patterns, the penetrance is estimated to be around 20% with variable expressivity in the disease presentation [5]. A further report indicated that while a 13.5% family history was present in patients with KC, phenotypes were not following true Mendelian inheritance patterns, thus indicating an incomplete penetrance and variable expression [34,44].

### 3.2. Syndromic KC

Although KC is frequently identified as an isolated ocular disorder, it has also been associated with a spectrum of systemic conditions, encompassing genetic, congenital, and acquired diseases [5]. Among systemic conditions, Down syndrome, Leber’s congenital amaurosis, atopy, and connective tissue disorders such as Marfan syndrome and Ehlers–Danlos syndrome have demonstrated the strongest syndromic associations with KC [5].

Down syndrome, resulting from trisomy of chromosome 21, has been widely associated with an increased incidence of KC. The prevalence of KC in individuals with Down syndrome is significantly higher than in the general population, with estimates suggesting up to a tenfold increased risk [45]. In pediatric populations with Down syndrome, the reported incidence of KC ranges from 0% to 32%, reflecting variability across cohorts and diagnostic criteria [46]. In contrast, up to 71.3% of adults with Down syndrome have been found to exhibit corneal topographic changes consistent with KC, underscoring a markedly increased prevalence with advancing age in this population [47]. Down syndrome patients are thought to be at higher risk due to higher rates of eye rubbing and alteration of collagen structure [48]. Atopy demonstrates a strong syndromic overlap with KC, primarily through its association with habitual eye rubbing and an enhanced inflammatory environment [49]. Eye rubbing, a common clinical manifestation in atopic individuals, is a well-established risk factor for KC and contributes to mechanical microtrauma of the cornea [50]. Additionally, the proinflammatory state inherent to atopic conditions, when combined with the repetitive mechanical stress of eye rubbing, further predisposes the cornea to the structural and biochemical changes characteristic of KC [50]. Connective tissue disorders, including Ehlers–Danlos syndrome, Marfan syndrome, and osteogenesis imperfecta, have also been syndromically associated with KC [51]. This relationship is thought to arise from underlying abnormalities in collagen cross-linking and broader connective tissue alterations, which may compromise corneal biomechanical integrity and predispose the cornea to the progressive thinning and ectasia characteristic of KC [50].

The strong familial pattern and syndromic causes observed in KC further incentivized research into genetic basis and studies, including linkage analyses, candidate gene studies, genome-wide association studies, and expression studies. Multiple genes, loci, and polymorphisms have been identified for KC.

### 3.3. Genes Affected in KC

Multiple genes are affected in KC resulting in a higher risk of developing the disease. Table 1 briefly summarizes the findings and variant classification of the main genes possibly altered in KC. 

#### 3.3.1. Visual System Homeobox 1 (VSX1)

*VSX1* (OMIM 605020) is located in chromosome 20p11-q11, and it is perhaps the most studied gene associated with KC [25,52]. Table 2 summarizes the information regarding *VSX1.* It has been shown to play a significant role in craniofacial and ocular development by regulating cone opsin expression during early stages of eye formation [53]. Although first linked to posterior polymorphous corneal dystrophy (PPCD), this gene has also been associated with KC, mainly through its role in eye development and its impact on stromal and retinal thinning [54,55,56].

Multiple variants of *VSX1* have been reported in patients with KC, including p.L17P, p.D144E, p.N151S, p.L159M, p.G160V, p.G160D, p.R166W, p.Q175H, p.H244R, and p.P247R [52,57]. One study in a population of Southern India failed to detect *VSX1* abnormalities in patients with KC, although it detected four SNPs: c.546A>G rs12480307, c.627+23G>A rs6138482, c.627+84T>A rs56157240, and c.504-24C>T (IVS3-24C) [58]. Another study in Saudi Arabia identified five SNPs related to *VSX1* and KC: g.8326G>A, g.10945G>T, g.11059A>C, g.5053G>T, and g.8222A>G [59]. In another study involving Chinese patients with sporadic KC, two *VSX1* variants, p.R131P and p.G160V, were identified in 3 out of 50 affected individuals and were absent when compared against a control group, suggesting a potential, though limited, association with disease susceptibility [60]. In addition, *VSX1* mutations were found in 5% of patients in Iranian and Italian cohorts [61]. Italian cohorts were further analyzed and discovered several SNPs, including p.L17P, p.D144E, p.H244R, p.P247R and p.G239R. In a New Zealand cohort, a novel mutation, c.173C>T, p.Pro58Leu was identified in a patient presenting with both KC and PPCD; however, subsequent replication studies were unable to detect this variant in additional control populations [62]. In an Italian patient, the *VSX1* mutation p.G239R c.715G>C was identified; this variant was absent in a control group of 200 individuals, suggesting a potential pathogenic role specific to KC [61]. *VSX1* has also been found to disrupt interactions between the complex protein network system of the eye, mainly between interactions within the collagen genes, MMP, and signaling pathways [53]. Nonetheless, replication studies have also had trouble replicating the findings of the *VSX1* relationship with KC in other studies [57,63,64]. A study group of English patients failed to identify *VSX1* in patients with KC [64]. No significant association was found in previous GWAS regarding KC and *VSX1* [65].

#### 3.3.2. Transforming Growth Factor Beta Induced (TGFBI)

*TGFBI* (OMIM 601692) is another candidate gene which codes specific pathways which alter corneal scar formations and fibrosis in wounds [66,67]. Located in chromosome 5q 31.1, it plays a role in modulating cell adhesion, movement, and interaction with the ECM in the stroma [68]. In 2017, Kabza and collaborators reviewed the role of *TGFBI* in KC and found that its downregulation accompanies collagen synthesis disruption and maturation [69]. *TGFBI* also plays a role in the secretion of the βig-h3 protein, which plays a further role in the corneal stroma modulating elastic fiber, fibronectin, and collagen type II [68]. More recently, a novel mutation of *TGFBI*, c.1406G>A, was detected in a family of Chinese patients with KC [70]. Another study determined a relationship between thyroxine levels and *TGFBI* expression in patients with KC while also affecting the prevalence of collagen types I and V, further determining a shift in the corneal ECM [71]. While the role of *TGFBI* in KC might not be entirely understood, another study group suggested the relationship of a sister gene, *TGFB2*, when they discovered it was overexpressed and had increased signaling in corneal epithelium in patients with severe KC [72].

#### 3.3.3. Zinc-Finger E Homeobox-Binding (ZEB1)

*ZEB1* is one of the two members of the ZEB gene family. Found in 10p11.22, this gene plays a pivotal role in developing the transition between epithelial and mesenchymal cells by inhibiting the expression of protein E-cadherin 1 (CDH1) [73]. *ZEB1* is abundantly present in the cornea’s epithelium basal membrane, vascular endothelial cells, and infiltrated immune cells [73]. Mutations in *ZEB1* have been shown to be implicated in endothelial dystrophies like PPCD and Fuchs Endothelial Corneal Dystrophy (FECD) [73]. Multiple variants were determined in a study by Lechner and collaborators in 70 patients with KC. These included mutations in exon 7 c.1920G>T and a missense *ZEB1* mutation in p.Gln640His. This study also demonstrated that several collagen genes (*COL4A1/2, COL4A3/4,* and *COL8A2*) were all downregulated in patients with KC [74]. Another study in a family with pediatric sporadic KC in Spanish patients found missense variants p.(Glu728Asp) [75]. Mutations in *ZEB1* also trigger binding of proinflammatory cytokines and upregulate their expression. While a direct link with KC has yet to be shown, its relationship in several patients affected with both PPCD and KC has been hypothesized to be linked [73].

#### 3.3.4. microRNA 184 (MIR184)

*MIR184* is a member of the microRNA family, a group of small non-coding RNAs that play key regulatory roles in gene expression by promoting mRNA degradation and suppressing translation. Previously, *MIR184* and mutations in its respective chromosomal region, 15q22-25, were linked to early-onset cataract cases [76]. Acting in conjunction with other regulatory RNAs, *MIR184* influences a variety of cellular pathways [77]. Notably, *MIR184* is the most abundant microRNA in the human cornea, with particularly high expression in the basal epithelial cells and corneal endothelium [77,78]. Dysfunction of *MIR184* impairs the repression of two key proteins, INPPL1 and ITGB4, both of which are thought to be involved in regulating corneal wound healing and structural maintenance [77,79,80]. These proteins promote epithelial adhesion and survival, and mutations in *MIR184* could further impair their protective role [81]. Mutations in *MIR184* have been reported in multiple families with KC, further supporting its potential role in disease pathogenesis [77,79,82]. A mutation in r.57C,T was identified in a patient from Ireland with familial KC and early-onset cataract across three generations [79]. Other substitutions included +3A → G and +8C → A and were found in patients with KC [82]. A larger study in Saudi Arabian patients indicated no *MIR184* mutations in 134 patients with KC, suggesting the mutation of *MIR184* is rare and more predominant in family cases [77]. While a single sporadic mutation in *MIR184* may not be sufficient to initiate the development of KC on its own, it can potentiate other predisposing factors that contribute to the disease’s onset and progression.

#### 3.3.5. Superoxide Dismutase 1 (SOD1)

*SOD1* was initially linked to Down syndrome due to their location on chromosome 21q22.11 [83]. The gene encodes an enzyme responsible for the dismutation of superoxide radicals, thereby playing a critical role in oxidative stress regulation. In the context of KC, mutations in *SOD1* were hypothesized to reduce enzymatic activity, leading to the accumulation of superoxide radicals. This oxidative imbalance may contribute to corneal damage, particularly through the formation of peroxynitrite within the corneal stroma [83]. It has also been demonstrated that *SOD1* is distributed in a different manner in keratoconic corneas when compared to healthy corneas [84]. The most common mutation for *SOD1* in KC is an intronic 7 bp deletion called c.169+50delTAAACAG, which generates nonfunctional *SOD1* proteins [53,83,84]. Although the mutation is estimated to have a low prevalence of approximately 2.6%, the substantial variability and inconsistency across studies suggest that this figure may not be entirely reliable [85]. Another study identified four nucleotide alterations (g.12035C4A, g.13978T4A, g.12037G4A, and g.11931A4C) in the *SOD1* gene among patients with KC, which were primarily classified as benign polymorphisms with no clear clinical relevance [86]. Nonetheless, studies in Iranian and Saudi Arabian cohorts have failed to link *SOD1* to KC patients in some cohorts [53,87]. Another Australian GWAS aimed to identify the presence of *SOD1* alterations in patients with KC but found no damaging *SOD1* variants in 385 patients [88]. To this day, it remains inconclusive whether *SOD1* plays a relevant part in the pathogenesis of KC.

#### 3.3.6. Zinc Finger 469 (ZNF469)

*ZNF469* is a two-exon gene located in 16q24, which encodes a 413 kDa protein of 3925 amino acid residues [89]. Table 3 briefly summarizes the information. It has been associated alongside *COL5A1* and *COL8A2* in several conditions, including brittle cornea syndrome (BCS), Ehlers–Danlos, and PPCD, and while the physiological role has not been entirely described, the evidence is that it regulates ECM and maintenance, potentially leading to dysregulation of the ECM when mutations occur [90]. *ZNF469* also contributes to the homeostasis of corneal fibers alongside 3 main types of collagens (*COL1A1*, *COL1A2*, and *COL4A1*) and to the transcription factors or extranuclear regulator factors in the human cornea [89]. As BCS is considered a connective tissue disorder with an autosomal recessive hereditary pattern and is associated with extreme corneal thinning, a link was investigated between *ZNF469* and KC [90]. Another closely related gene, *PRDM5*, has been associated with BCS and has also been linked to KC, even affecting patients at a younger age compared to controls [90,91]. A theory proposed shows that *ZNF469* could be related to alterations in the transforming growth factor beta (TGFβ) pathway [89].

An early study detected 14 rare missense variants of *ZNF469* in 46% of patients from Polynesian and Māori descendant patients [92]. Further studies in a Han Chinese population indicated 7 mutations in *ZNF469*, indicating a possible pathological significance [89]. A study by Lechner and collaborators identified allele mutations in 12.5% of patients with KC [90]. Another missense variant p.Arg492Gln was recently located in a family of Spanish descent [75]. Another Spanish family with KC was identified for variations in c.2972del, p.Pro991Hisfs62 of *ZNF469* [93]. A further expression analysis of keratoconic corneas demonstrated underexpression of *ZNF469* alongside other ECM genes. This suggests a reduced collagen regulation and impaired ECM remodeling in keratoconic corneas [28]. Recently, a GWAS study determined a significant association between SNPs rs2721051 and rs9938149 in sporadic KC associated with mutations in both *ZNF469* and *FOX1* genes [94].

A study conducted in a Polish cohort found no significant enrichment of sequence variants in *ZNF469*, with the identified changes classified as common polymorphisms in the general population [95]. These findings are consistent with results from an Australian patient cohort, in which the observed *ZNF469* variants were similarly determined to have no association with KC susceptibility [96]. Even so, other studies have shown to link *ZNF469* variants in patients with advanced KC [97].

#### 3.3.7. Lysyl Oxidase (LOX)

*LOX*, a copper-dependent enzyme, functions by activating collagen cross-linking and elastin by catalyzing oxidative deamination in certain hydroxylysine residues [98,99]. *LOX* catalyzes the oxidation of epsilon amino groups in peptidyl lysines to reactive aldehydes, which subsequently undergo spontaneous condensation with other ε-amino or aldehyde groups. This process facilitates the cross-linking of collagen and elastin, ultimately converting them into insoluble, stable ECM fibers [100]. Deficiency in *LOX* genes has been found in other connective tissue disorders like cutis laxa, Ehlers–Danlos, and Menkes [98].

Earlier studies identified a significant linkage peak at the 5q23.2 locus, implicating *LOX* along with neighboring genes as potential contributors to KC susceptibility. Subsequent GWAS analysis observed association at SNPs rs10519694 and rs2956540 located within intron 4 of *LOX*, which contribute to KC by family association, and polymorphisms in two exons of *LOX*: rs1800449 and rs2288393 [98]. Similarly, an Iranian cohort of 112 patients with KC, which aimed to investigate the latter two polymorphisms, found a significant difference with rs1800449 when compared to control groups [101]. A meta-analysis demonstrated a significant association between SNPs rs10519694 and rs2956540 in patients with KC but failed to demonstrate sufficient evidence to demonstrate association with the two known polymorphisms rs1800449 and rs2288393 [102]. A recent Chinese cohort identified an alteration on c.95G>A as a possible altered gene in *LOX* in patients with KC [103].

Expression studies show *LOX* transcript levels are typically reduced in patients with KC, indicating diminished enzymatic activity in their tear film [28]. Moreover, a study by Shetty et al. demonstrated that decreased *LOX* expression correlates with disease severity and is accompanied by altered expression of COL1A1 and COL4A1, genes critical to collagen synthesis and corneal structure [100]. *LOX* has long been considered one of the most promising genes to understand the pathogenesis of KC [65].

#### 3.3.8. Dedicator of Cytokinesis 9 (DOCK9)

*DOCK9* is a gene which is responsible for encoding protein families that possess GTP/GDP exchange factors and activates G-protein CDC42 [25,55]. It was first established as a possible risk factor for KC by mutations in the linkage locus 13q32 following an autosomal dominant model [25,104]. Aberrations in c2262A>C, which in turn provides substitutions in *DOCK9*, were also reported to be present in KC patients in a cohort of patients from Ecuador, generating a polymorphism in rs7995432 [105,106]. Mutations have also been identified in two additional genes associated with the *DOCK9* pathway: *IPO5* (*Importin 5*) and *STK24* (*Serine/Threonine Kinase 24*), both of which are expressed in the human cornea. This disrupted the *DOCK9* mutation, leading to exon 20 skipping altered proteins [65,106].

Nonetheless, further studies need to be implemented to determine the causal factor between *DOCK9* and KC. A study in a Brazilian population aimed to determine the relationship between rs7995432 in *DOCK9* but found no significant difference when compared to a control group [107].

#### 3.3.9. Sodium Bicarbonate Trasporterlike Protein 11 (SLC4A11)

*SLC4A11*, located on chromosome 20p13, encodes a member of the bicarbonate transporter superfamily, SLC4, which functions as an electrogenic sodium-coupled borate cotransporter. Loss or dysfunction of this transporter has been shown to induce apoptotic pathways, contributing to cellular degeneration [108]. Located in the corneal epithelium and endothelium, functional impairment of *SLC4A11* leads to oxidative stress, mitochondrial dysfunction, and corneal edema in mouse models [109]. Mutations in *SLC4A11* have been found in patients with congenital hereditary endothelial dystrophy type 2 (CHED2) and have also been linked to Fuchs endothelial corneal dystrophy (FECD) and KC [110]. A substitution and deletion of *SLC4A11* in c.2558+149_2558+203del54, along with a substitution of another gene, IL1RN, was detected in an Ecuadorian family to be significantly different when compared to non-KC family members [108]. Another proband missense variant p.Gly769Arg was found in a Spanish family with Honduran ancestry [75]. More recently, mutations in the *SLC4A11* gene have been reported in individuals with KC among Chinese families in conjunction with other candidate genes [111]. Among these, *SLC4A11* and *TGFBI* have demonstrated the most significant genetic upregulation between KC and non-KC patients, further supporting their potential role in disease susceptibility [28]. As with other genes, it is possible the main role of *SLC4A11* in KC is still unknown, and further studies are required.

#### 3.3.10. Tissue Inhibitor of Metalloproteinase 3 (TIMP3)

*TIMPs* are recognized as the endogenous regulators of matrix metalloproteinases (MMPs), protecting tissue from active irreversible destruction [103]. A tightly controlled balance between *TIMPs* and MMPs is essential for maintaining ECM homeostasis, governing critical biological processes such as embryonic development, tissue remodeling, wound healing, and morphogenesis [61]. Initial studies showed the expression and linkage of *TIMP3* to stromal corneal cell apoptosis and their presence in corneas with KC [112,113]. An initial study by Bonis et al., aiming to screen for multiple gene alterations in patients with KC, which included *TIMP3*, failed to discover pathogenic *TIMP3* mutations, only determining several SNPs which were evenly distributed also in controls [61,85]. Recent novel mutations in *TIMP3* c.476C>T have been identified in Chinese patients with KC, suggesting a potential role in ECM dysregulation. These alterations may affect ECM integrity either through *TIMP3* overexpression, leading to increased apoptotic activity in the corneal stroma, or through underexpression, impairing the tissue’s ability to resist injury-related degradation [103]. A GWAS failed to relate previously AMD-related genes to KC when considering gender and age as covariables, although expressed interest was marked for SNP rs5749482, which could be linked to KC [114]. Other expression studies have identified the underexpression of *TIMP3* in KC corneas, which leads to heightened ECM degradation [115]. Recently, another study identified a reduced level of tissue metalloproteinase inhibitors in blood serum, heightening the important diagnostic value of *TIMP3* and suggesting a possible systemic component to its downregulation [116].

#### 3.3.11. Interleukin 1 Alpha (IL1A)/Interleukin 1 Beta (IL1B)/: Interleukin 1 Receptor Antagonist (IL1RN)

*IL1A* and *IL1B* codify IL-1a and IL-1b, proinflammatory cytokines responsible for inflammatory, immune, and hematopoiesis responses. While *IL1RN* codifies for the receptor antagonist, which modulates the response to these proinflammatory cytokines [108]. These genes, located at locus 2q13-q14.3, play important roles in the inflammatory pathways by developing B cells, inducing secretion of IL-6 by T cells, and stimulating the release of further proinflammatory cytokines like IFN-γ and tumor necrosis factor (TNF).

A polymorphism of *IL1A*, rs2071376, was noted in a Korean study. These findings suggested that this polymorphism could play a minor protective role in KC [117]. Two polymorphisms of *IL1B* genes, rs16944 and rs1143627, were associated with greater risk of KC in a Korean population [108,118]. This association was subsequently replicated in a Japanese cohort, where the rs1143627 polymorphism in *IL1B* demonstrated a statistically significant correlation with increased KC risk, further reinforcing its potential role as a genetic susceptibility marker in diverse populations [119].

Similarly, keratocyte apoptosis has been reported in up to 60% of KC patients triggered by the epithelial release of IL1 after mechanical injury of the epithelium [118]. IL1 proteins enhance the expression of keratocyte-derived products, including collagenases and matrix metalloproteinases (MMPs), which subsequently facilitate corneal wound healing and tissue remodeling following injury [118,120,121]. Similarly to other genes, these genes were also found to be absent in several populations; more specifically, a IL1β-511C>T and *IL1RN* VNTR were studied and determined that they do not play a part in the development of KC in a Turkish population [122].

#### 3.3.12. Collagen Type IV Alpha 3 Chain (COL4A3)/Collagen Type IV Alpha 4 Chain (COL4A4)/Collagen Type V Alpha 1 Chain (COL5A1)

Corneal stroma is formed predominantly by collagen, which gives the tissue structure, shape, and strength. All three genes are related to the adequate onset of the corneal collagen structure during embryogenesis. *COL5A1* encodes an alpha chain subunit of collagen type V, abundantly predominant in the corneal stroma [123,124].

Collagen genes have long been implicated in KC. In Slovenia, 3 *COL4A3* variants—P141L, D326Y, and G895G—and 5 *COL4A4* variants—P482S, M1327V, V1516V, and F1644F—were significantly differentiated in the KC groups [125,126,127]. Furthermore, in an Iranian population, a *COL4A4* polymorphism of rs2229813 (M1327V) allele A was found to be significant in developing KC [128]. A meta-analysis determined that two *COL4A4* SNPs demonstrated strong associations with KC in white populations: rs2229813 and rs2228557. Yet the same meta-analysis determined that both SNPs were not associated in non-white cohorts [127].

Initial evidence demonstrated that mutations in *COL5A1* are associated with corneal thinning, a hallmark feature of KC [123]. A missense variant, c.1372C>T, was identified in patients with KC in a Chinese cohort [103]. A subsequent study conducted in a Russian cohort reinforced the genetic significance of three SNPs—rs1536482, rs2721051, and rs1324183—which showed a strong association with KC susceptibility. In contrast, two rare promoter variants (rs1043208782 and rs569248712) were identified in only two affected individuals from a single family, suggesting that such rare variants are unlikely to contribute substantially to the overall genetic risk of KC [129]. The role of *COL5A1* mutations in KC is further strengthened by the novel discovery of acceptor-splice mutations found in Indian patients [124]. Recently, the presentation of a patient with KC and pectus excavatum was reported with mutations in *COL5A1*, further hypothesizing the role of *COL5A1* in altering collagen pathways in the cornea [130]. *COL4A3* and *COL4A4* have been identified as absent and not at risk for KC in a Greek population [131]. *COL5A1* has also been found to not play a major role in KC development in Russian cohorts [129].

**Figure 3:**
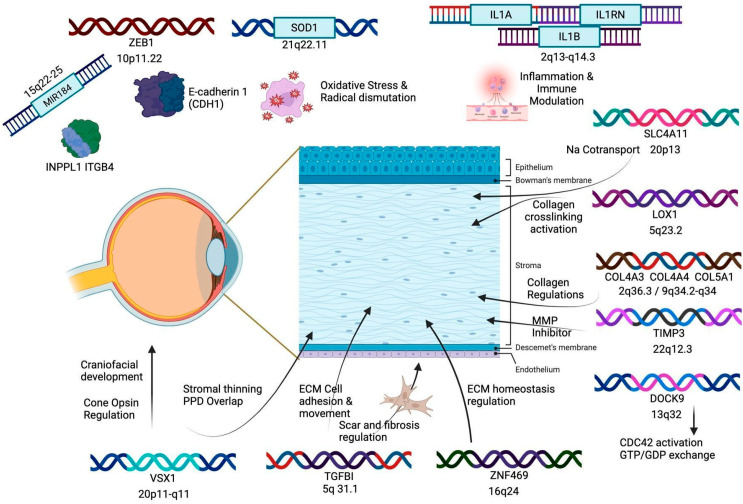
Altered genes and their clinical implications in KC. **Note:** KC emerges from a multifactorial imbalance including genetic factors. Alterations in diverse genes can create changes in the eye or the cornea at a structural or molecular level.

Figure 3 visually describes the clinical implications of the altered genes in KC while Table 4 summarizes the findings in Section 3 of all genes discussed above.

## 4. Future Directions in Genetics and KC

Genetic testing in KC has advanced drastically in the last decade. New advances in technology, gene sequencing, and GWAS have provided in-depth knowledge on many KC genes, locations, and polymorphisms. Future directions include expanding research to larger and more diverse GWAS cohort groups, including multi-ethnic population studies. Another major objective would be the integration with transcriptomics, proteomics, and epigenomics to further map the regulatory networks involved in KC. With the advancements of artificial intelligence, the use of deep learning machines can correlate genetic sequences and profiles to further understand the relationship with disease. The main goal of understanding genetics is to provide better treatment and prevention goals. Currently no gene therapy for KC exists. Future advancements could bring the possibility of enabling disease-modifying therapy to the disease via therapeutic gene therapy.

## 5. Conclusions

KC remains a multifactorial corneal ectasia in which genetic determinants play a key pivotal role in the disease. Advancements in candidate gene studies, linkage analysis, and GWAS have identified multiple variants associated with ECM integrity, oxidative stress, and inflammatory regulation, reinforcing the genetic contribution to the disease pathogenesis. However, much remains uncertain. Reports vary and are specific to certain populations; causality is often unproven, and the functional consequences of most associations are still poorly defined. Future research should prioritize large, multicentric, and multiethnic cohorts; functional validation; and integration of genetic data with biomechanical and environmental risk factors. These approaches will not only refine diagnostic and predictive models but also lay the foundations for therapeutic strategies for the future of KC.

## Figures and Tables

**Table 1:** Genes in KC.

Gene Name	Loci	Variant Classification
*VSX1*	20p11-q11	Variant of Uncertain Significance
*TGFBI*	5q 31.1	Variant of Uncertain Significance
*ZEB1*	10p11.22	Variant of Uncertain Significance
*MIR184*	15q22-25	Some Pathogenic (regarding syndromic KC) Some Variants of Uncertain Significance
*SOD1*	21q22.11	Variant of Uncertain Significance
*ZNF469*	16q24	Some Pathogenic
		Some Variants of Uncertain Significance
*LOX*	5q23.2	Variant of Uncertain Significance
*DOCK9*	13q32	Variant of Uncertain Significance
*SLC4A11*	20p13	Variant of Uncertain Significance
*TIMP3*	22q12.3	Variant of Uncertain Significance
*IL1A/IL1B/IL1RN*	2q13-q14.3	Likely Bening
*COL4A3/COL4A4/COL5A1*	COL4A3: 2q36.3; COL4A4: 2q36.3; COL5A1: 9q34.2-q34.	Some Pathogenic Some Variants of Uncertain Significance

**Table 2:** *VSX1* in KC: genetic variants, biological functions, and population evidence.

Location	Chromosome 20p11–q11
Biological Role	Regulates cone opsin expression during early eye development, particularly in the corneal stroma and retina
Functional Impact	Disrupts protein interaction networks, particularly collagen, MMPs, and signaling pathways
Associated Conditions	BCS, Ehlers–Danlos, PPCD
Reported Variations	p.L17P, p.D144E, p.N151S, p.L159M, p.G160V, p.G160D, p.R166W, p.Q175H, p.H244R, p.P247R, p.R131P, p.G239R c.715G>C, p.Pro58Leu c.173C>T
Population-Specific Findings	Southern Indians: No mutations; 4 SNPs detected: rs12480307, rs6138482, rs56157240, IVS3-24C Saudi Arabians: 5 SNPs linked: g.8326G>A, g.10945G>T, g.11059A>C, g.5053G>T, g.8222A>G Chinese: Variants p.R131P and p.G160V in 3/50 patients, absent in controls Iranian and Italian: Mutations in ~5% of patients; SNPs: p.L17P, p.D144E, p.H244R, p.P247R, p.G239R New Zealanders: Novel c.173C>T p.Pro58Leu in KC + PPCD patient, not replicated England: No association found

**Table 3:** ZNF469 in KC: genetic variants, biological functions, and population evidence.

Location	Chromosome 16q24
Biological Role	Regulates ECM maintenance and corneal fiber homeostasis
Functional Impact	Suggested role in ECM remodeling and collagen remodeling
Associated Conditions	BCS, Ehlers–Danlos, PPCD
Reported Variations	14 rare missense variants in Polynesian/Māori (46% of patients) 7 mutations in Han Chinese patients Allele mutations in 12.5% of KC patients p.Arg492Gln in Spanish family c.2972del, p.Pro991Hisfs62 in another Spanish family
Population-Specific Findings	Polynesian/Māori: High frequency of rare variants (46%) Han Chinese: 7 mutations detected Spanish: Two families with rare variants European: 12.5% of patients carried variants Polish: No enrichment, changes were polymorphisms Australian: No significant association Advanced KC patients: Variants linked in some studies

**Table 4:** Brief summary of genes, variations, and alterations reported in patients with KC.

Gene	Location	Main Function	Variations Reported	Potential Phenotypical Impact	Populations Described
*VSX1* [52,53,54,56,57,58,59,60,61,132]	20p11-q11	Cone opsin regulation, craniofacial regulation	p.L17P, p.D144E, p.N151S, p.L159M, p.G160V, p.G160D, p.R166W, p.Q175H, p.H244R, p.P247R, c.546A>G, p.R131P, p.L17P, p.D144E, p.H244R, p.P247R, g.8326 G>A, g.10945 G>T, g.11059 A>C, g.5053 G>T, g.8222 A>Gp.G239Rp.G160V rs12480307, c.627+23G>A rs6138482, c.627+84T>A rs56157240, c.504-24C>T IVS3-24C, c.173C>T p.Pro58Leu, p.G239R c.715G>C	Stromal thinning, PPCD overlap	Present in Saudi Arabian [59], Indian [58], Chinese [56,60], Iranian [53], Caucasians [61], Italian [132], New Zealanders [62] Absent in English [64], Korean [63], Greek [133], Saudi Arabian [59] (Inconclusive), American [57]
*TGFBI* [66,67,68,69,70,71,72]	5q 31.1	Modulates scar formation, fibrosis. Regulates cell adhesion, movement, and interaction in the ECM.	c.1406G>A	Decreased βig-h3 protein, fibrosis susceptibility, stromal ECM alterations	Present in Polish [69], Chinese [70], German [71] Absent in European populations [134], Chinese [135]
*ZEB1* [73,74,75]	10p11.22	Regulates the expression of protein E-cadherin 1 (CDH1)	exon 7 c.1920G>T, missense *ZEB1* mutation in p.Gln640His, p.Glu728Asp	PPCD overlap	Present in Spanish [75], Caucasian [74], Chinese [61] Absent in Chinese and Greek [66]
*MIR184* [76,77,78,79,80,81,82]	15q22-25	miRNA regulation of INPPL1 and ITGB4 proteins	r.57C,T, +3A>G, +8C>A	Early onset cataracts overlap familial KC	Present in Irish [79], Saudi Arabians [77] Absent in Iranian [82]
*SOD1* [53,61,83,86,87,133,136]	21q22.11	Manages oxidative stress regulation by dismutation of radicals	c.169+50delTAAACAG, g.12035C4A; g.13978T4A; g.12037G4A g.11931A4C	Oxidative stress imbalance	Present in Caucasian [83] Absent in Saudi Arabian [86], Brazilian [84], Australian [88]
*ZNF469* [89,90,92,93,94,95,96,97]	16q24	ECM regulation, collagen maintenance, homeostasis of collagen fibers	p.Arg492Gln, rs2721051, rs9938149, c.2972del, p.Pro991Hisfs62	BCS overlap	Present in Polynesian and Māori [92], Spanish [75,93], Caucasian [90] Absent in Polish [95], Australian [96], Saudi Arabian [137]
*LOX* [98,99,100,101,102]	5q23.2	Activation of collagen cross linking by catalyzing oxidative deamination	Rs10519694, rs2956540, rs1800449, rs2288393,	Potential stromal weakening	Present in Iranian [102], Chinese [103], Caucasian [32] Absent in Brazilian [61]
*DOCK9* [104,105,106,107]	13q32	GTP/GDP exchange factor, CDC42 activation	c2262A>C, rs7995432	Protein dysregulation	Present in Ecuadorians [105,106] Absent in Polish [105], Brazilian [107]
*SLC4A11* [103,108,109,110,111]	20p13	Electrogenic Na+-coupled borate cotransporter	C.2558+149_2558+203del54, p.Gly769Arg	Induced apoptotic pathways, contributing to cellular degeneration, oxidative stress, mitochondrial dysfunction, and corneal edema	Present in Ecuadorians [108], Spanish [75], Chinese [103,111]
*TIMP3* [112,113,115,116]	22q12.3	Endogenous MMP inhibitor	c.476C>T	ECM degradation imbalance, stromal apoptosis	Present in Chinese [103] Absent in Italian [114], Brazilian [85]
*IL1A/IL1B/IL1RN* [117,118,119,120,121]	2q13-q14.3	Inflammatory cytokines, immune modulation	Rs2071376, rs16944, rs1143627	keratocyte apoptosis, ECM remodeling	Present in Korean [108,118], Japanese [119] Absent in Turkish [122]
*COL4A3/COL4A4/COL5A1* [123,124,125,126,127,128,129,130]	*COL4A3*: 2q36.3; *COL4A4*: 2q36.3; *COL5A1*: 9q34.2-q34.	Collagen network formation and regulation in corneal stroma	P141L, D326Y, G895G, P482S, M1327V, V1516V, F1644F, rs2229813, rs2228557, c.1372C>T, rs1536482, rs2721051, rs1324183, rs1043208782, rs569248712	Corneal collagen alterations	Present in Chinese [103], Caucasian [123,125,126,127], Indian [124], Iranian [128] Absent in Greek [131], Russian [129], Pakistani [138]

## Data Availability

No new data were created or analyzed in this study.

## Abbreviations

BAD	Belin Ambrosio Display
BCS	Brittle Cornea Syndrome
CBI	Corvis Biomechanical Index
CHED2	Congenital Hereditary Endothelial Dystrophy
COL4A3	Collagen Type IV Alpha 3 Chain
COL4A4	Collagen Type IV Alpha 4 Chain
COL5A1	Collagen Type V Alpha 1 Chain
DOCK9	Dedicator of Cytokinesis 9
FECD	Fuchs Endothelial Corneal Dystrophy
GWAS	Genome Wide Association Studies
IL1A	Interleukin 1 Alpha
IL1B	Interleukin 1 Beta
IL1RN	Interleukin 1 Receptor Antagonist
KC	Keratoconus
LOX	Lysyl Oxidase
MIR184	microRNA 184
PPCD	Posterior Polymorphous Corneal Dystrophy
SLC4A11	Sodium Bicarbonate Transporter Like Protein 11
SNP	Single Nucleotide Polymorphism
SOD1	Superoxide Dismutase 1
TBI	Tomography and Biomechanical Index
TGFBI	Transforming Growth Factor Beta Induced
VSX1	Visual System Homeobox 1
ZEB1	Zinc-Finger E Homeobox-Binding
ZNF469	Zinc Finger Protein 469
